# The UCSF Mouse Inventory Database Application, an Open Source Web App for Sharing Mutant Mice Within a Research Community

**DOI:** 10.1534/g3.120.401086

**Published:** 2020-03-09

**Authors:** Estelle Wall, Jonathan Scoles, Adriane Joo, Ophir Klein, Carlo Quinonez, Jeffrey O. Bush, Gail R. Martin, Diana J. Laird

**Affiliations:** * Department of Obstetrics, Gynecology and Reproductive Science; Center for Reproductive Sciences; Eli and Edythe Broad Center for Regeneration Medicine and Stem Cell Research, University of California; † San Francisco, CA 94143; ‡ Program in Craniofacial Biology and Department of Orofacial Sciences, Univeristy of California, San Francisco, CA 94143; § Department of Pediatrics and Institute for Human Genetics, University of California, San Francisco, CA 94143; †† Department of Cell and Tissue Biology; Program in Craniofacial Biology; Institute for Human Genetics, University of California, San Francisco, CA 94143; ‡‡ Department of Anatomy, University of California, San Francisco, CA 94143; ** ThermoFisher Scientific, Carlsbad, CA 92008

**Keywords:** mouse, inbred strain, transgene, mutant allele, web application

## Abstract

The UCSF Mouse Inventory Database Application is an open-source Web App that provides information about the mutant alleles, transgenes, and inbred strains maintained by investigators at the university and facilitates sharing of these resources within the university community. The Application is designed to promote collaboration, decrease the costs associated with obtaining genetically-modified mice, and increase access to mouse lines that are difficult to obtain. An inventory of the genetically-modified mice on campus and the investigators who maintain them is compiled from records of purchases from external sources, transfers from researchers within and outside the university, and from data provided by users. These data are verified and augmented with relevant information harvested from public databases, and stored in a succinct, searchable database secured on the university network. Here we describe this resource and provide information about how to implement and maintain such a mouse inventory database application at other institutions.

Following the development of methods for obtaining germline transmission of mouse gene knock-outs created by homologous recombination in mouse embryonic stem (ES) cells (Thomas and Capecchi 1987; Thompson *et al.* 1989), there clearly arose a need for a central, online catalog for the mouse genome, mutants and their phenotypes. This led to the creation of the Mouse Genome Database by the Mouse Genome Informatics (MGI) Group in 1994. In 2007, organizations collaborated to form the International Knockout Mouse Consortium (IKMC) using a high-throughput approach to target the mouse genome in ES cells. Today, over 50,000 mutant alleles are listed in the Mouse Genome Database, and a large increase in this number is anticipated as endonuclease-mediated technologies such as CRISPR/Cas9 are becoming routine (Bult *et al.* 2019). Many mutants can be obtained from commercial vendors or repositories, or requested from other investigators, but imports can be time-consuming and costly; frozen embryos or sperm require cryo-resuscitation, whereas transfer of live mice involves testing for pathogens, quarantine, and often rederivation. Particularly at large institutions, investigators may not be aware of the mutant mice that exist within their home facility, which can lead to duplicative import of lines.

The UCSF Mouse Inventory Database Application (hereafter known as the Mouse Database Web App) has been in continuous operation for 10 years, with records for more than 2700 different mutant alleles, transgenes, and inbred strains maintained by investigators at UCSF. The Web App was conceived and established by Dr. Gail Martin in 2009 as a shared resource to enable members of the community of mouse researchers to find out what mutant mice are housed at UCSF, reduce the costs associated with animal purchases and transfers from external sources, promote collaboration, support grant-writing and pilot studies, and promote knowledge about and the use of official mutant mouse nomenclature. The database is readily available through a web interface and yet limited to the university community. Labs voluntarily list mice to be shared upon request for no cost beyond the internal transfer, with the expectation that their mouse records are kept private within the university. To achieve these benefits, the Mouse Database Web App requires financial and administrative support, technical support in setting-up the open-source platform, researcher engagement, and cooperation with the animal care facility, in order to maintain accuracy and promote usage.

Numerous researchers at other institutions, including many who trained or worked at UCSF, have made inquiries about how to establish such a shared catalog at their current institutions. The following sections describe the user experience of the UCSF Mouse Database Web App and provide information about what is involved in establishing an equivalent resource at any research facility.

## Methods

### Features of the mouse database web app and user experience

The organizing principle is that each entry (record) in the database is given a unique number and describes an individual mutant allele, transgene, or inbred strain. These three categories are defined below.

#### Mutant allele:

has one or more specific modifications to a locus within the genome that can result in amorphic, hypomorphic, hypermorphic, antimorphic, or neomorphic changes to function. Mutations may involve a single nucleotide change or replacement of stretches of several kilobases using genetic engineering technologies, such as homologous recombination. With the advent of targeted endonucleases, including CRISPR/Cas9, TALENs, and ZFN technology, endonuclease-mediated (em) has been added to the mutant mouse nomenclature. Em technologies can be used to create single or multi-locus modifications for traditional knockout or knock-in mutations or to perturb promoter and exon regions for interference or activation (Knowlton and Smith 2017).

#### Transgene:

contains a fragment of DNA (from mouse or another organism) inserted at a random location in the genome, usually as concatemers. The most common method for generating transgenes is by injection of a DNA fragment into a fertilized egg (pronuclear injection into the zygote), but these can also be produced using ES cell engineering. In recent years Next Generation Sequence technology has accelerated the ability to identify transgene integration sites and MGI has updated official symbols to reflect these identified loci; for example *Tg(ACTB-cre)2Mrt* is now *Tmem163^Tg(ACTB-cre)2Mrt^*.

#### Inbred strain:

is homozygous at all loci and individuals are genetically identical. Inbred strains result from at least 20 generations of brother-sister inbreeding. Many inbred strains that were established decades ago carry QTL and spontaneous mutations that are increasingly being identified. For more information, see https://www.jax.org/jax-mice-and-services/customer-support/technical-support/genetics-and-nomenclature/inbred-mice.

### Information Included in a database mouse record

**Figure 1:**
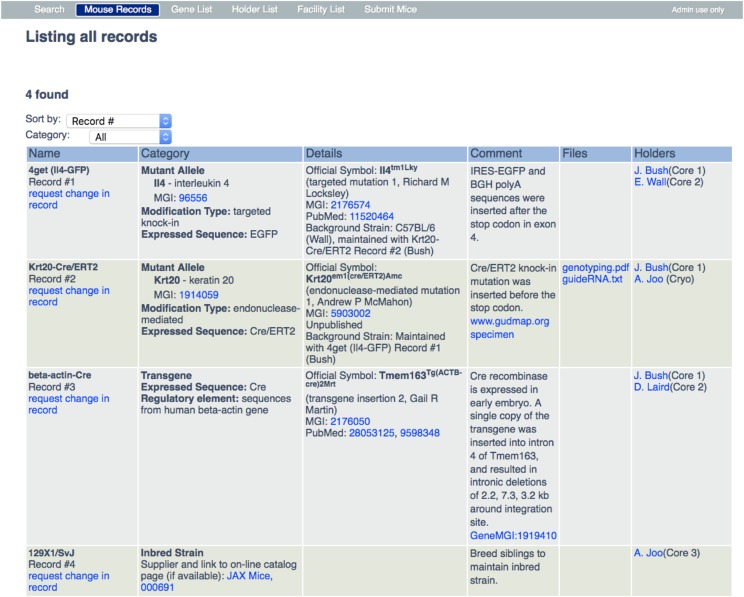
Sample mouse records table. A view of the Mouse Record page in the Database Web App shows examples of records for each of the three categories in which entries are classified: mutant allele, transgene, inbred strain. A full description of the information in each column is provided in the section of the text entitled “Information included in a Database Record”; here attention is drawn to the hyperlinks in the records (shown in blue): In the “Name” column, every record has a link to a change request form that can be completed online. The “Category” column in the records for mutant alleles (records #1 and #2) contains links to the page on MGI that describes the gene that is altered in the mutant allele. In record #4 there is a link to the supplier’s catalog page for the inbred strain listed. The “Details” column provides links to the MGI and PubMed accession pages for the mutant alleles and transgenes. The “Comments” column provides some information about modifications with additional relevant URL links. The “Files” column contains links to downloadable files for genotyping protocols and oligonucleotide sequences. The “Holders” column has a link to the E-mail address for each Holder in order to facilitate communication with the holder about the entry. The following are examples of the URLs for the hyperlinks. MGI:96556, http://www.informatics.jax.org/marker/MGI:96556, MGI:2176574, http://www.informatics.jax.org/allele/MGI:2176574, PubMed: 11520464, https://www.ncbi.nlm.nih.gov/pubmed/11520464?dopt=Abstract, www.gudmap.org specimen, https://www.gudmap.org/chaise/record/#2/Gene_Expression:Specimen/RID=N-GZ5G.

The database in the Web App is designed to include essential information about each entry. Sample database records for two mutant alleles (records #1, 2), a transgene (record #3) and an inbred strain (record #4) are shown in Figure 1.

The “Name” column in a record gives a name (sometimes a nickname) by which the mouse line is known to the investigator(s) at UCSF who is(are) maintaining it (termed “holder[s]”) and a permanent, unique record number for it. Users can ‘request change in record’ by filling out the linked form.

The “Category” column describes the kind of modification. For mutant alleles, the official symbol for the gene that is mutated as well as its MGI ID number are provided. In addition, the type of modification that was made to that gene is described, based on criteria established by MGI http://www.informatics.jax.org/userhelp/ALLELE_phenotypic_categories_help.shtml (Eppig 2017). Here it is noted when the modification includes expression of an introduced sequence such as a EGFP. For transgenes, the expressed sequence (*e.g.*, Cre) and regulatory elements it contains are described. For inbred strains the name of the supplier and catalog number is provided.

In the next column, essential “Details” are supplied for each entry. For mutant alleles and transgenes, the official MGI-designated symbol is provided, along with its accession number in the MGI database (http://www.informatics.jax.org). In addition, the PubMed ID for the first publication describing the generation of the mutant allele or transgene is listed. This information is obtained from MGI, which curates information on mouse mutants from ∼12,000 primary research publications annually (Bult *et al.* 2019). There is also a field for background strain information if the mutant allele or transgene described in the record is maintained on an inbred background (Record #1).

It is very important to note that investigators often maintain a given mutant allele or transgene in combination with other mutant alleles or transgenes. Because each record describes an individual mutant allele or transgene, this issue is addressed by including information about the combination in the “Background strain” field. This information includes the name(s) and record number(s) of the other mutant alleles/transgenes present (see Details column in Figure 1, Record #1, 2).

The “Comment” column provides relevant information such as a brief description of the structure of the mutant allele or its phenotype. If a Material Transfer Agreement is required, this is stated here. For mutations that are not listed in MGI and are unpublished, the donating lab provides these details. Many members of our research community list their engineered mice before publication and use the Mouse Database Web App as a channel for dissemination and collaboration.

The “Files” column provides downloadable pdf, csv or text files that contain genotyping protocols, sequence information, or background breeding information regarding the mutant allele or transgene.

The “Holders” column lists the Principal Investigator(s) in whose mouse colony the mutant allele, transgene, or inbred strain is currently maintained and the facility in which it is housed (*e.g.*, Core 1). To make it easy to request mice, the PI name(s) in this column are linked to a blank E-mail form with the name of the mutant allele, transgene, or inbred strain and its database record number in the subject line. The E-mail is addressed to the PI or a person who has been designated to handle such requests.

### Database web app homepage and navigation

**Figure 2:**
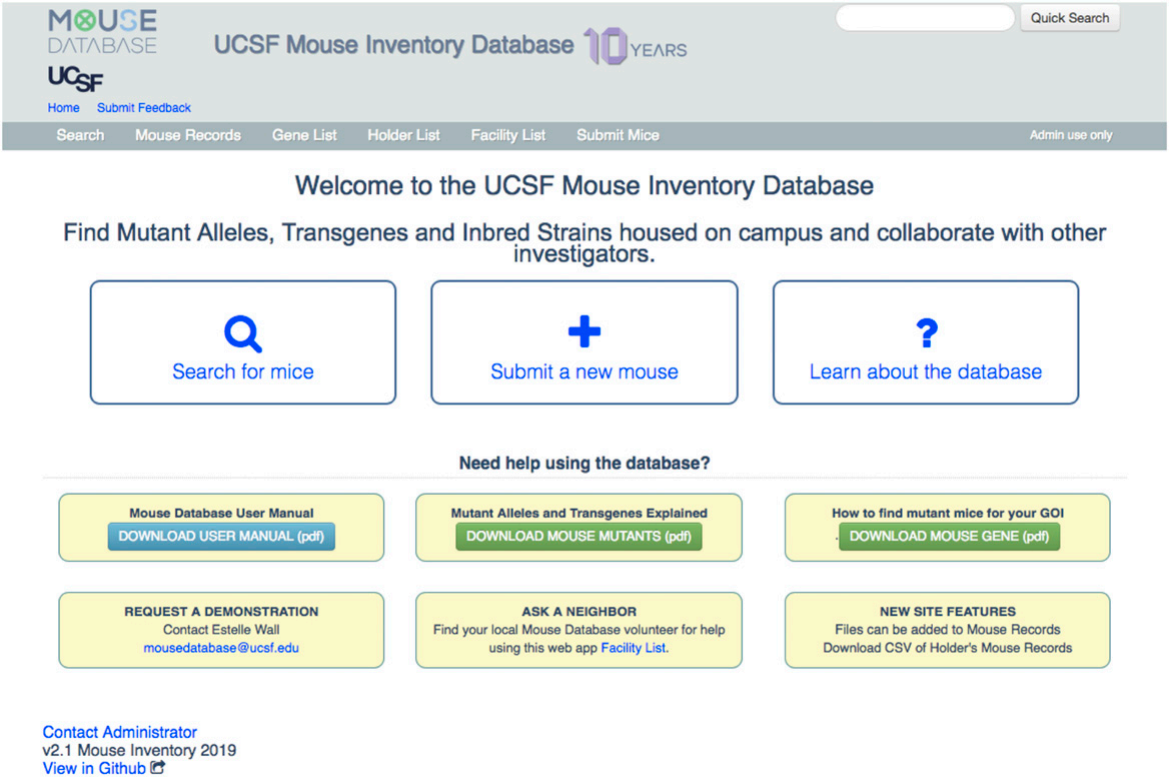
The Mouse Database Web App Homepage. The Navigation Bar across the top has links (in white lettering) to specific pages (see Figure 3). The three large boxes provide links to (left) the database search engine, (middle) a form for requesting the creation of a new record, and (right) a detailed description of all the features of the Database Application and a list of Frequently Asked Questions (FAQs). The “Quick Search” field (top right) routes to the search results page (see Figure 3A). The yellow boxes provide resources for education about mouse mutants and means of getting help in using the Application.

**Figure 3:**
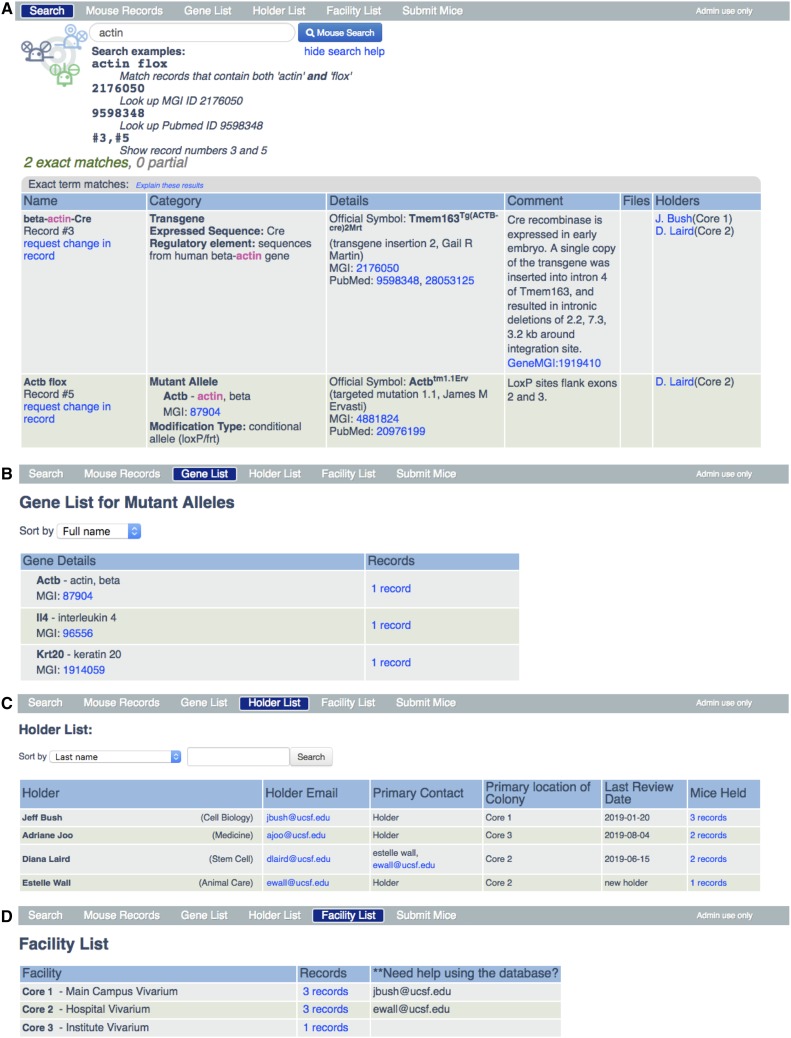
The navigation bar. A demo database was created with 5 mouse records, 4 holders, and 3 facilities for the purpose of displaying the various features of the mouse database application. (A) Clicking on the *Search* button brings up a search field page and when a search term is entered (in this case ‘actin’) redirects to a search results page like the one shown here. It includes the search field at the top with the search term that was entered followed by sample queries (“Search examples”). Below that, all of the records that contain the search term are displayed. (B) *Gene List* enables users to sort the genes that are modified in mutant alleles and includes links to the mouse record table for those mutations. (C) *Holder List* is a searchable table of principal investigators with contact details and links to their mouse records and when records were audited (last review date). (D) *Facility List* describes all the vivaria, each of which has a link to a display of the mouse records table for all the mice housed in that facility. The names of individuals who use that facility and are willing to provide help in using the Web App are provided. Clicking on *Submit Mice* brings up the online form for requesting the creation of a new record. Clicking on *Admin use only* (extreme right of the navigation bar) provides access to the password-protected administrator portal (see Figure 4).

The Mouse Database Web App search engine is accessible as a web interface to any individual using a university IP address or virtual private network (VPN) to help ensure that the information is restricted to those within the university. The home page (Figure 2**)** includes three boxes that contain links to key features: searching for mice, adding new mice, and getting help. The search function enables one to call up browsable lists that include: all records; all genes mentioned in one or more records; all PIs whose mice are listed in the database (holders); and all mutant alleles, transgenes, or inbred strains housed in each separate campus vivarium. Users can search by specific words in mouse records, gene names, record numbers, MGI ID, PubMed ID, or holder. For example, a search for “actin” (Figure 3A) would bring up all records currently in the inventory with names that include “actin,” such as a β-actin conditional null allele, or a β-actin-Cre transgene. The “Submit a new mouse” box (Figure 2) links to a form for requesting the creation of a new record. Educational information and opportunities to connect with the mouse community are offered in the six yellow boxes of the home page.

A navigation bar at the top of all pages in the Web App primarily provides quick access to major blocks of information in the database application. “Search,” (Figure 3A) links to the same page as the “Search for mice” box on the home page. “Gene List” (Figure 3B) provides a list of all loci targeted in mutant alleles. It can be sorted by full name, official symbol, or MGI number, and – in the records column - links to a display of all records in which that gene is mutated (not shown). “Holder List” (Figure 3C) provides the names of all principal investigators whose mice are listed in the database (Holders) with contact information, including - when designated - the name and contact information for the person who serves as the Primary Contact for that Holder’s laboratory. Other information listed here includes the campus and building in which the mouse colony for that PI is located, and a link to all the records in the database on which that holder is listed. Finally, “Facility List” (Figure 3D) shows all the different mouse facilities at the University, with a link to all the records for mice held in that facility. Importantly, the records for a specific facility can be sorted by “Mouse Name,” Record number, or category (*i.e.*, mutant allele, transgene, or inbred strain).

### System overview and application code

**Figure 5:**
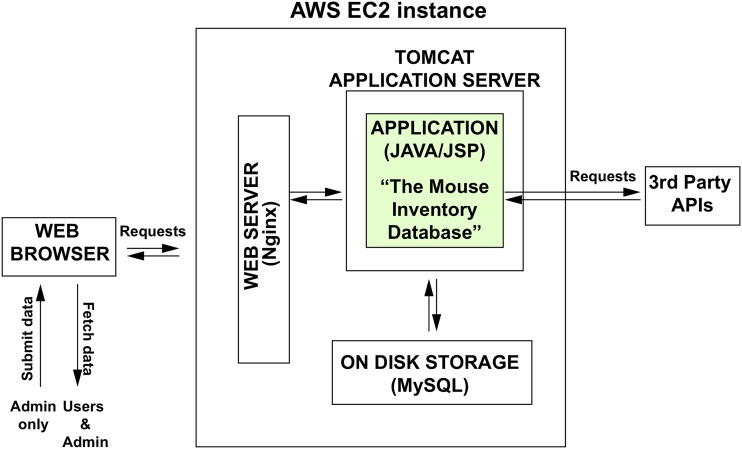
System overview of the Mouse Inventory Database Application. Users and admin access the Database Web App via a Web Browser (*e.g.*, Safari, Chrome, Firefox, etc.). The Web Browser contacts the Web Server, which runs on an Elastic Compute Cloud (EC2) instance hosted in Amazon Web Services (AWS). The Web Server functions to ensure the security of the web connections. The Web Server proxies the request to a Tomcat Application Server, which routes the request to the “Mouse Inventory Database” Application, which is coded in Java, using JSP, JDBC, and additional custom logic. Data processed by the Application is stored in a MySQL database. The Application also makes requests to 3^rd^ Party Application Programming Interfaces (APIs) such as Mouse Genome Informatics (MGI) and PubMed. Such requests enable the database to automatically look up and include important information about the mutant alleles and transgenes: their accession number in the MGI database and the PubMed ID for the first publication describing how each was produced.

A schematic overview of the design of the Database Web App is shown in Figure 5. The Mouse Database Application was written in Java EE and the information is stored in a MySQL or MariaDB database with code and deployment instructions being freely available. The application can be deployed and viewed by designated users within a virtual private network (VPN) using an inexpensive hosting service such as Amazon Web Services (AWS) or Microsoft Azure. The Administrator controls all changes to the database, and works through the Web App to upload data in csv format and E-mail users using a semi-automated E-mail template system. To facilitate the harvest of data from MGI for integration into mouse records (such as MGI accession numbers and Pubmed IDs), the application code has been configured with a free developer’s account to access the MGI Public Ad Hoc SQL server http://www.informatics.jax.org/software.shtml#sql. A downloadable demo application with recent code updates is available on GitHub at our repository develop branch (https://github.com/musIndex/mouseinventory/tree/develop), which can be run from the command line and viewed locally in a browser using instructions in File S3.

### Data availability

The Mouse Database Web App source code along with instructions for deploying the platform are publicly available on GitHub (https://github.com/musIndex/mouseinventory). Version 2.1 develop branch code and a demo database were used to generate figures and is available at https://github.com/musIndex/mouseinventory/tree/develop. Supplemental material consisting of user and administrator manuals and source code README file areavailable at figshare: https://doi.org/10.25387/g3.11852550.

## Results and Discussion

### Database content

**Figure 4:**
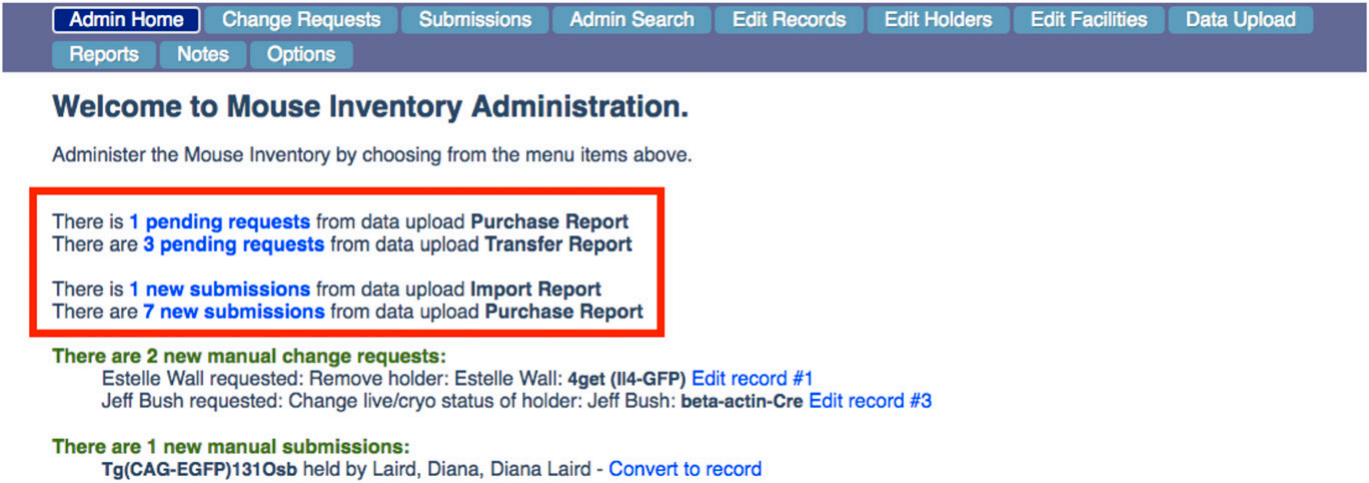
Administrator Navigation Portal. The navigation bar for the Admin Portal is shown at the top with a view of the Admin Home Page below. This page provides the Administrator with a to-do list for keeping the database up-to-date. The red box contains a list of action items generated by the semi-automated system that reports purchases and transfers from the animal care facility: 1 purchase and 3 internal transfers are in process for mice already in the database and these “pending requests” need to be pursued to determine if changes to the existing records need to be made (*e.g.*, adding new holders); purchases of 7 mice and 1 import from another university are in process for mice not in the database. These “new submissions” need to be pursued to determine if new records need to be added. In addition, requests submitted by users online for changes to 2 existing records and a new *submission* are in the queue to be edited. Other buttons in the navigation bar enable the Administrator to access pages that contain information on Change requests or Submissions, or to Edit Records, Edit Holders. The Admin Search item button enables the Administrator to view all mouse records including records that are screened from view by users (*e.g.*, records that have been deleted from the database). The Data Upload and Reports buttons provide access to features of the semi-automated system. The Notes button provides access to a page where the Administrator can create and edit panels for admin view only. *Options* gives access to pages for editing the content panels on the Main Home Page and for storing templates for emails sent to holders by the Administrator.

The utility of the database depends on its content being accurate and up to date through active engagement between users and the Administrator. The database is populated with new mice through the user submitted form, Submit Mice tab on the navigation bar (Figure 3), and edited by the Administrator who commits the new mouse records to the database through the Administrator portal (Figure 4). The Administrator is the sole person who modifies the database using the Administrator portal. Users submit recommended changes to existing records, such as adding or deleting a holder via a link (“request a change in record”) in the “Name” column (Figure 1). This is the only method by which the information that a holder is no longer maintaining a particular mutant allele, transgene, or inbred strain is transmitted to the Administrator. A second channel for adding mice and adding holders to mouse records comes from the Administrator batch processing data of mouse purchases from vendors, transfers within the university and imports from other universities. These data are used by the Administrator to create and edit records as well as to contact users using E-mail templates (File S2). These user requests, new mouse submissions, and data uploads are stored in the Administrative section of the database, which is accessed by a password-protected “Admin use only” link on the extreme right of the Navigation Bar (Figure 3).

The Administrator curates the database records, drawing from a working knowledge of mouse genetics and familiarity with the research community. Although the database is designed such that there can be only one mouse record for a mutant allele or transgene with a given MGI accession number, the Administrator verifies that new records without assigned MGI accession numbers do not already exist in the database. In addition, the Administrator confirms the information imported from the MGI database by checking that the referenced PubMed ID describes the generation of that specific mutant allele or transgene and uses this reference to annotate the ‘Comment’ section of the record with information about gene element modification, the inserted cassette, tissue-specific expression, etc. The Administrator also adds relevant URLs and upload files. For MGI accession numbers, the official symbol may change to reflect new gene names, especially in the case of transgenes and in such cases it is necessary for the Administrator to input these changes. If an MTA was required for transferring mice to the University, then a holder choosing to list the mouse in the database is responsible for informing any requestors; this information is included in the Comment section on the mouse record. The administrator adds user submitted background strain breeding and genotyping files, however validation and quality control of mice happens between collaborating laboratories. To ensure accuracy in holder mouse lists, the administrator performs an annual audit with holders and researchers.

Promotion and education around the database within University community is critical to its success. A brief user manual with guidelines is made available on the Mouse Database Web App (File S1) along with FAQs. The Administrator conducts outreach to new faculty or their lab managers individually as well as providing an annual demonstration of the database to incoming graduate students. Security and trust are built through broadcasting the limited access to the Web App and the expectation that users maintain privacy of holder information. In summary, the Administrator plays a central role in engaging the mouse user community, ensuring the accuracy of information in more than 2700 mouse records, and communicating with over 300 holders.

### Resources needed to establish and maintain the mouse database application

After a decade of continuous operation, the UCSF Mouse Database Web App has nucleated collaborations and pilot studies, saved time and research funds, fostered a spirit of cooperation, and left its imprint on a generation of trainees and faculty. Our community members report using the Web App frequently, as often as weekly, and in particular when preparing grant proposals and designing new experiments. This resource is also a tool to help investigators catalog the mutations and maintain genotyping protocols for their own mouse colonies. Accordingly, Google analytics reported an average of 4,600 user sessions annually for the Web App from 2017 through 2019.

To understand what is needed to establish the equivalent of the UCSF Mouse Database Web App at another institution, it is helpful to know how it came to be established at UCSF. The Web App was conceived, designed, and implemented by Dr. Gail Martin working closely with Jonathan Scoles, a professional software engineer. Initially, persuading members of the UCSF community to participate in the Web App enterprise was accomplished by Martin making contact with individual faculty colleagues and then giving presentations about the Web App to research groups. Once numerous faculty had become participants and the value of the database had become evident, other avenues such as E-mail campaigns targeting faculty with active mouse protocols were conducted. Thus, the chances of successfully implementing an equivalent Web App at another institution will be greatly improved if there is one or more faculty members who are deeply committed to leading the effort to recruit labs to participate in and manage the overall function of the database.

Another requirement is a database Administrator and a means to cover the cost. Currently, at UCSF the Web App Administrator dedicates 50% effort to curation as well as outreach. Two faculty members serve as advisors to the Administrator and liaisons with the IACUC. A budget of ∼$50,000 per year to support the salary of the Web App Administrator, nominal fees for web services, emergency software repairs and improvements is included in animal care costs. The usage, including website visits, numbers of mouse records and holders is tracked and reported to the animal oversight committee each year. By far the biggest challenge in establishing the Web App at UCSF was that many researchers could not precisely identify which mutant alleles and transgenes they were maintaining. Education outreach is an important part of the Administrator’s job, in order to inform users about mouse gene nomenclature, the MGI database, and how to properly characterize mice in the 3 categories (mutant allele, transgene, inbred strain).

### Perspective and future directions

Despite the success of the UCSF Mouse Database Web App there are ongoing challenges. Continued funding depends on the support of the local animal care facility and ultimately the investigators who work with mice, hence communication and responsiveness to users is crucial. Maintaining an up-to-date database is a continual challenge, as is obtaining user responsiveness to E-mail queries.

One very important issue is the accuracy of the records in the database. This can be problematic when the Web App is first implemented at an institution because investigators who have been maintaining their mice for long periods may no longer have accurate information about which specific mutant allele of a particular gene or which version of a particular transgene they had acquired. This is not a problem when the mice are purchased from commercial vendors, from repositories, or imported from the investigators who produced the mutant alleles or transgenes and the records of these acquisitions have been maintained by the animal care facility. The movements of mice are documented by the Web App through recording all user ‘change request’ forms, ‘mouse submission’ forms, and administrator-uploaded data. In the future we plan to indicate in the record which method was used to acquire the mouse, track changes in background breeding and include genotyping protocols to help address this issue.

Another important issue, which has become increasingly clear, is that the genetic background and level of inbreeding of the strains used in experiments can have a great impact on the interpretation and reproducibility of results (Dietrich *et al.* 1993; Threadgill *et al.* 1995). An accessible and widely available tool for assessing the purity of genetic background is the Mouse Universal Genotyping Array (MUGA) (Welsh *et al.* 2012; Collaborative Cross Consortium 2012), which is used to validate all mouse strains archived and distributed by the Mutant Mouse Resource and Research Centers (MMRRC). The ‘Background Strain’ field under Details in the mouse record should be used to state background composition, while the file attachment can be used to document breeding schemas and share genotyping methods. Despite efforts to relay accurate information, users of the Mouse Database Web App are ultimately responsible for authenticating the mice they receive from holders.

Although the content of the Mouse Database Web Application is available only to the UCSF community, our goal is to share the tools for setting up a similar resource in other organizations. To that end, we have provided the Database Application code on the Github repository (https://github.com/musIndex/mouseinventory). Ongoing updates to the code as well as a demo database SQL file can be found under the develop branch with instructions for development and implementation (https://github.com/musIndex/mouseinventory/tree/develop). If developers wish to extend the functionality, forking the repository in Github allows their improvements to be merged upstream so as to benefit the entire community. Questions, suggestions, or problems regarding the code can be posted by the public on the ‘issue tracker’ for our repository at https://github.com/musIndex/mouseinventory/issues. We strive for active engagement of the broader mouse user community in using and modifying the code for the benefit of all. The code is sufficiently versatile to be developed for other shared resources such as cataloging other research organisms or reagents. In our efforts to develop the code and provide instructions on working with it, we hope this manuscript will engage the broader research community to post on our GitHub issue tracker and branch our code for further development. In disseminating information about how the UCSF Mouse Inventory Database Application is constructed, maintained and modified as an institutional mouse database, we hope to further enhance its functionality and in turn improve access, knowledge, and collaboration among the mouse research community and beyond.
